# Supplementary figures and images for: A prognostic model for predicting the duration of 20,049 sickness absence spells due to shoulder lesions in a population-based cohort in Sweden
Source: PLoS One. 2023 Jan 20;18(1):e0280048. doi: 10.1371/journal.pone.0280048 (PMC9858371; doi:10.1371/journal.pone.0280048)

**Supporting information: Calibration plots of binary outcomes SA > 90, 180 and 365 days respectively.
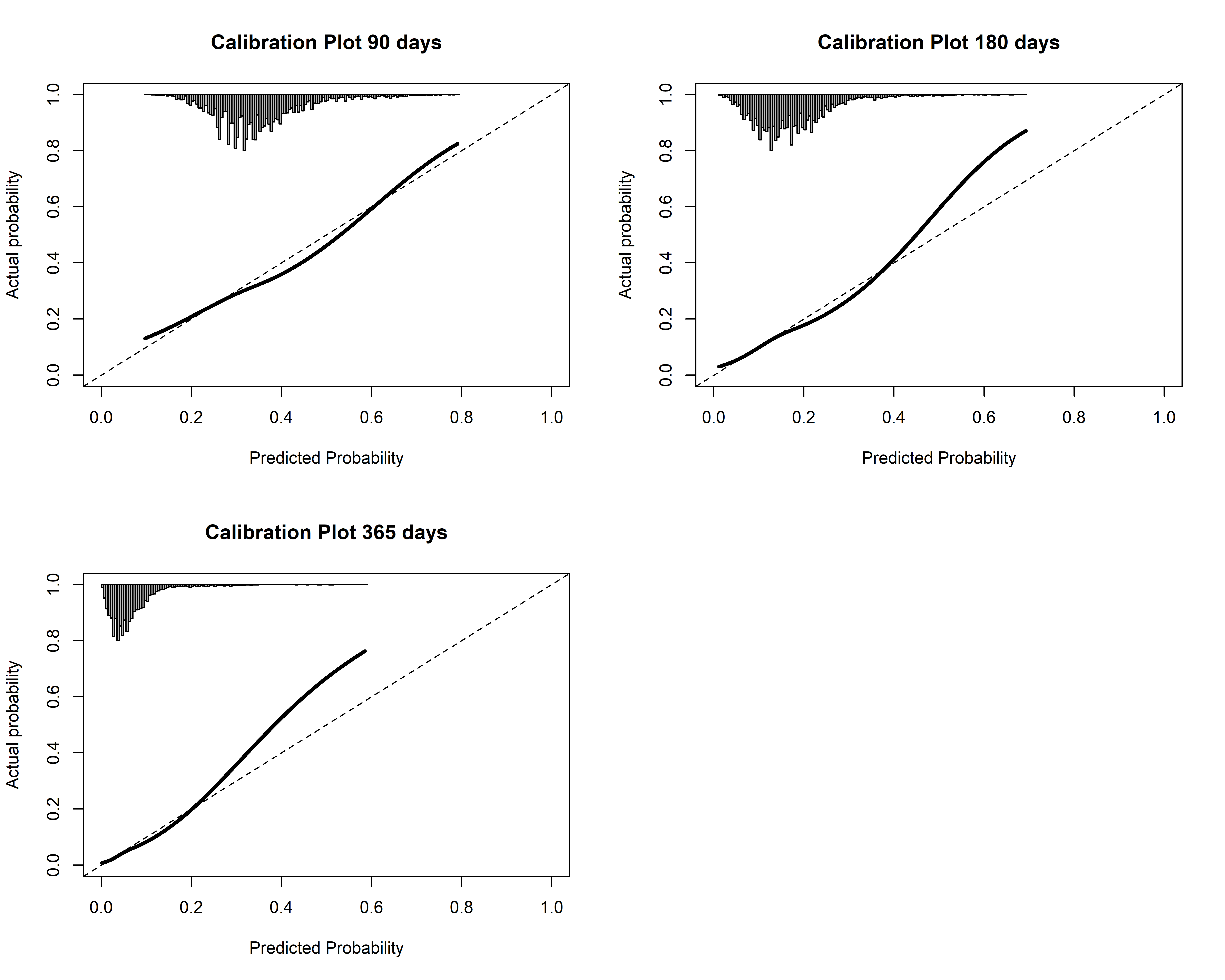
**

Supplement: S1 Fig — (DOCX) [file pone.0280048.s001.docx]
