# Supplementary material for: A prognostic model for predicting the duration of 20,049 sickness absence spells due to shoulder lesions in a population-based cohort in Sweden
Source: PLoS One. 2023 Jan 20;18(1):e0280048. doi: 10.1371/journal.pone.0280048 (PMC9858371; doi:10.1371/journal.pone.0280048)
Supplement: Attachment — Submitted filename: revision_1216.docx [file pone.0280048.s003.docx]

Manuscript number PONE-D-22-17574R1

Title: A prognostic model for predicting the duration of 20,049 sickness absence spells due to shoulder lesions in a population-based cohort in Sweden

Dear Editor Kjell Torén,

# Thank you very much for the opportunity to submit a revised version of this manuscript! We appreciate reviewer #2’s additional valuable comments as well as the comments from you and have addressed them point by point below. We have also revised the manuscript accordingly, using the Track-Changes tool.

# On behalf of all authors, yours sincerely,

# Katalin Gemes

**Comments from reviewer #2**

Thank you for the opportunity to review this interesting and timely paper from Gemes at al. It is a nice study dealing with a prognostic model for predicting the duration of 20,049 sickness absence spells due to shoulder lesions in a population-based cohort in Sweden. A prediction model of the duration of sickness absence (SA) spells due to shoulder lesion was developed, with ease of clinical implementation in mind. Discriminatory ability was poor in the short term but improved for predicting very long durations of SA. The model could be useful for prognostications of duration of SA spells and can provide information regarding which cases that might be in need of extra rehabilitation measures to promote return to work.

The study is solidly performed, and the topic is relevant, and as the authors point out, there is currently lack of scientific knowledge in the area.

**Author’s response:** Thank you for these positive comments.

There are some areas that require attention, and these are noted below:

“1. Work ability depends on the individual's resources but also on the physical and psychosocial demands of the work. The LISA database provides access to occupational codes. Have the authors included, for example, job sector or occupation as a predictor? If not, I suggest that the authors discuss why and raise this in the discussion section.”

**Editor’s comment**: “Please discuss the handling of the occupational codes.”

**Author’s Response:** Different occupational aspects were considered at the preselection phase as possible predictors. Those were: type of work (white or blue collar), type of work sector (private, public), and work situation at the start of the SA spell (in paid work, on parental leave, studying, unemployed) – of those three, only the last one had strong enough predictive value to be included in the analyses. This is not what we had expected- as your comment suggests, one would have expected that at least blue/white collar would be predictive – why we of course checked those analyses extra many times. Due to these results, we did not study specific occupations.

Probably the type of occupation has more impact on the risk of becoming sickness absent; not on the duration of SA spells among those who actually are on SA. The same goes e.g., for sex; women have a higher risk for becoming sickness absent, however, among those sickness absent, women in general have the same risk of long-term SA spells.

We now added more information about this in the Method section:

*(Some of the variables that in the initial process did not show strong enough predictive value to be included among those 14, were two of the work related: namely type of sector /private vs. public) and type of work (blue vs. white collar)).* (page 7)

**Reviewer’s comment 2**: “The authors have used ICD10-code M.75, and as they describe, they do not have access to more detailed information with the subgroups that exists. Regarding sickness absence, it is for example probably more likely that a person with adhesive capsulitis has a longer sickness absence than some of the other diagnoses in this group. I suggest that the authors clarifying that problem in the discussion section.”

**Editor’s comment: 2.** “Have you more detailed information about the diagnosis than M75? Probably not, but please comment this in the Discussion part.”

**Author’s response**: We, of course, agree with the reviewer and Editor that the future work capacity as well as the SA prognosis might differ between M75 sub diagnoses within this group, as it would do with level of severity and/or type of treatment within each of those sub groups. Unfortunately, we didn’t have more detailed information on the SA diagnosis, nor on disease severity or on type of treatments. We now revised the limitation text as follows:

*We had no information on disease severity, nor on type of treatment (e.g., surgery or not), nor information on specific SA diagnostic M75 sub groups (we only had information about the three-digit level ICD-10 code from MiDAS). Such types of information could have improved the discriminatory ability of the model and would be preferable to include in future studies [6].* (page 12)

**Reviewer’s comment:** 3. “In Sweden, the National Board of Health and Welfare has a sick leave support for shoulder problems, which is there to help the physicians to predict how long a person with a certain diagnosis is recommended to be on sick leave. Could this have influenced the outcome and be a predictor for how long the people were on sick leave? It would be helpful if this also was raised in the discussion section.”

**Editor’s comment 3**: “Please discuss the sick leave guidelines from the National Board of Helth and Welfare, and if those guidelines may have affected the length of the sick leaves.”

**Authors’ response:** There are, of course, many aspects that influence the duration of a SA spell. In Sweden, in the last decades, a large number of nationwide interventions were introduced in order to influence SA, due to the very high SA rates at the turn of the century. Some of those that had been introduced at the time of these SA spells are: interventions to increase to competence in insurance medicine among physicians and among social insurance officers, competence developments of healthcare CEOs regarding their management of how sickness certification of patients is handled in their clinic, stricter time limits regarding when a SA spell is to be checked by the social insurance officer (those are obvious in Figure 1, that is, at day 90, day 180, 365, and 914 of a SA spell), that social insurance officers to a larger extent also did check this (that is, a change in practice), more scientific knowledge about possible ‘side affects’ of being sickness absent among both claimants and professionals. Another of those interventions was the one you mention; that the National Board of Health and Welfare’s in 2007 introduced nationwide general recommendations for handling of sickness certification and for possible duration of sickness absence due to specific diagnoses.

The recommendations ‘only’ cover the most common SA diagnoses, and thus not all sub groups of M75. Those four subgroups that were covered at this time, all together under the same headline in the recommendations, were M75.0 (frozen shoulder), M75.1 (rotator cuff injuries), M75.3 (calcific tendinitis), and M75.4 (impingement). The recommendations stated that:

-M75.0; up to 2 weeks, if physically demanding job up to three weeks. After surgery: return to work (RTW) within a few days, of not physically demanding work. Otherwise, physically demanding job SA up through a month.
-M75.1. up to six weeks, if physically very demanding, up to 26 weeks.
-M75.3: up to 2 weeks, if physically demanding job up to three weeks.
-M75.4: up to 2 weeks, if physically demanding job up to three weeks. After surgery, SA up to three weeks if not physically demanding job, if physically very demanding job up to 12 weeks.

Moreover, in those recommendations it is clearly stated that the need for SA is to be individually assessed, and can vary widely for people with the same diagnoses. For instance, for the M75 diagnoses, the need might be shorter if not the dominant arm is affected. However, other aspects, such as multimorbidity, might lead to need of longer duration. Altogether, less than 20% of the M75 SA spells exceed the longest recommended time (26 weeks).

It is, of course, an interesting study question whether those recommendations influenced the duration of SA spells due to M75, however, such a study would have required both other data and another study design. Moreover, it would not be easy to differentiate effects from those recommendations from effects of the other interventions.

We have now, nevertheless, included a paragraph about this at the end of the Discussion section and also added two references regarding the recommendations for SA duration.

*In Sweden, several nationwide interventions have been introduced in the decade before those SA spells begun, such as interventions to increase competence in insurance medicine among physicians and social insurance staff, stricter time lines regarding when to assess claimants right to prolonged the SA spell (e.g., at day 90, 180, 365, and 914, as can be seen in Figure 1), and in 2007, recommendations from the Board of Health and Welfare regarding sickness certification and duration of SA spells due to specific diagnoses, including M75, namely due to M75.0 (frozen shoulder), M75.1 (rotator cuff injuries), M75.3 (calcific tendinitis), and M75.4 (impingement) [35, 36]. The degree to which those interventions might have had an impact on how long the SA spells became would require other types of studies and data.* (page 13)
